# Morphological characteristics of the Lisfranc ligament

**DOI:** 10.1186/s13047-020-00412-0

**Published:** 2020-07-16

**Authors:** Y. Suzuki, M. Edama, F. Kaneko, M. Ikezu, K. Matsuzawa, R. Hirabayashi, I. Kageyama

**Affiliations:** 1 Institute for Human Movement and Medical Sciences, Niigata University of Health and Welfare, Niigata, Japan; 2 Department of Anatomy, School of Life Dentistry at Niigata, Nippon Dental University, Niigata, Japan

**Keywords:** Lisfranc ligament, Plantar ligament, Lisfranc joint injury, Gross anatomy

## Abstract

**Background:**

This study aimed to clarify the morphological characteristics of the Lisfranc ligament and the cuneiform 1-metatarsal 2&3 plantar ligament (CMPL).

**Methods:**

Forty legs from 20 cadavers were examined. Classification proceeded according to the number of fiber bundles in the Lisfranc ligament and the CMPL. Morphological features measured were fiber bundle length, width, thickness, and angle.

**Results:**

In Type I-a, the Lisfranc ligament and the CMPL were a single fiber bundle; in Type I-b, the Lisfranc ligament was a single fiber bundle, and the CMPL was two fiber bundles; in Type II-a, the Lisfranc ligament was a two fiber bundle, and the CMPL was a single fiber bundle; in Type II-b, the Lisfranc ligament and the CMPL were two fiber bundles; in Type III-a, the Lisfranc ligament was three fiber bundles, and the CMPL was a single fiber bundle; in Type III-b, the Lisfranc ligament was three fiber bundles, and the CMPL was two fiber bundles; in Type IV, the Lisfranc ligament and the CMPL could not be separated. Type I-a was seen in 37.5%, Type I-b in 10%, Type II-a in 30%, Type II-b in 7.5%, Type III-a in 7.5%, Type III-b in 2.5%, and Type IV in 5%. The Lisfranc ligament was significantly larger than the CMPL in total fiber bundle width, total fiber bundle thickness, and total fiber bundle angle.

**Conclusion:**

The Lisfranc ligament had up to 3 fiber bundles and the CMPL had one or two fiber bundles; classifications were four types and two subgroups.

## Background

A Lisfranc injury is defined as any bony or ligamentous injury that involves the tarsometatarsal joints of the foot [1]. Lisfranc injuries are infrequent, accounting for approximately 0.2% of all fractures [2]. The calculated incidence of Lisfranc joint injuries was reported to be 1/88,000 new cases per year in New Zealand [3]. However, it has been estimated that up to 20% of Lisfranc injuries are misdiagnosed or missed altogether on initial evaluation [4]. It is essential to know and understand the anatomy of the tarsometatarsal joint (Lisfranc joint) to achieve a correct diagnosis and provide proper treatment of the injuries that occur at that level [5].

Injury to the Lisfranc joint can result from either a direct or indirect mechanism [6]. An indirect mechanism is more common and is usually from axial loading or twisting on a plantarflexed foot; direct Lisfranc injuries are less common and occur when a direct load is applied to the Lisfranc joint [7]. The Lisfranc joint includes all articulations between the tarsal bones (3 cuneiforms and the cuboid) and the bases of the 5 metatarsals. There is considerable inherent osseous stability, with the recessed second metatarsal (M2) base functioning as the integral keystone. As a result of this stability, the midfoot is rigid. The Lisfranc ligament proper is a thick oblique ligament extending from the base of M2 to the plantar aspect of the first cuneiform (C1). The integrity of this ligament is important for stability at the tarsometatarsal articulation, since there is no transverse metatarsal ligament between the first metatarsal (M1) and M2 [1]. Furthermore, in a biomechanical study using fresh-frozen cadavers [8], amputation of the Lisfranc ligament and the cuneiform 1-metatarsal 2&3 plantar ligament (CMPL) [9] was necessary to cause instability of the Lisfranc joint (C1-M2 joint and second cuneiform-M2 joint). Therefore, these two ligaments are important for the stability of the Lisfranc joint.

The ligamentous structures of the Lisfranc joint are the dorsal ligament, the interosseous ligament (the Lisfranc ligament), and the CMPL [5, 10, 11]. In anatomical studies, consistent findings have not been obtained for the morphological features of the Lisfranc ligament and the CMPL. There are reports that the Lisfranc ligament has a single fiber bundle [10], two fiber bindles [12], and four fiber bundles [11]. There is also large variation among reports on fiber bundle length, ranging from 8.02 mm to 33.7 mm [13–15]. The fiber bundle width was 2.53–12.5 mm [13–15], and bundle thickness was 5.4–7.68 mm [13–16]. The CMPL runs from the plantar surface of the C1 to the M2 and the third metatarsal (M3). Its directionality varies, and it divides into three directions depending on ligament morphology [11]. Therefore, the morphological characteristics of the two ligaments involved in the stability of the Lisfranc joint have not been sufficiently studied. This is one of the factors that makes it difficult to make a definitive diagnosis of Lisfranc joint injury.

Therefore, this study aimed to clarify the morphological characteristics of the Lisfranc ligament and the CMPL.

## Methods

### Cadavers

This investigation examined 40 legs from 20 Japanese cadavers (mean age at death, 81 ± 9 years; 22 sides from men, 18 from women; 20 right sides, 20 left sides) that had been switched to alcohol after placement in 10% formalin. None showed signs of previous major surgery around the foot or ankle or any relevant deformities, and there was no obvious degeneration in all specimens. This study was approved by the Ethics Committee at our institution.

## Methods

**Fig. 1:**
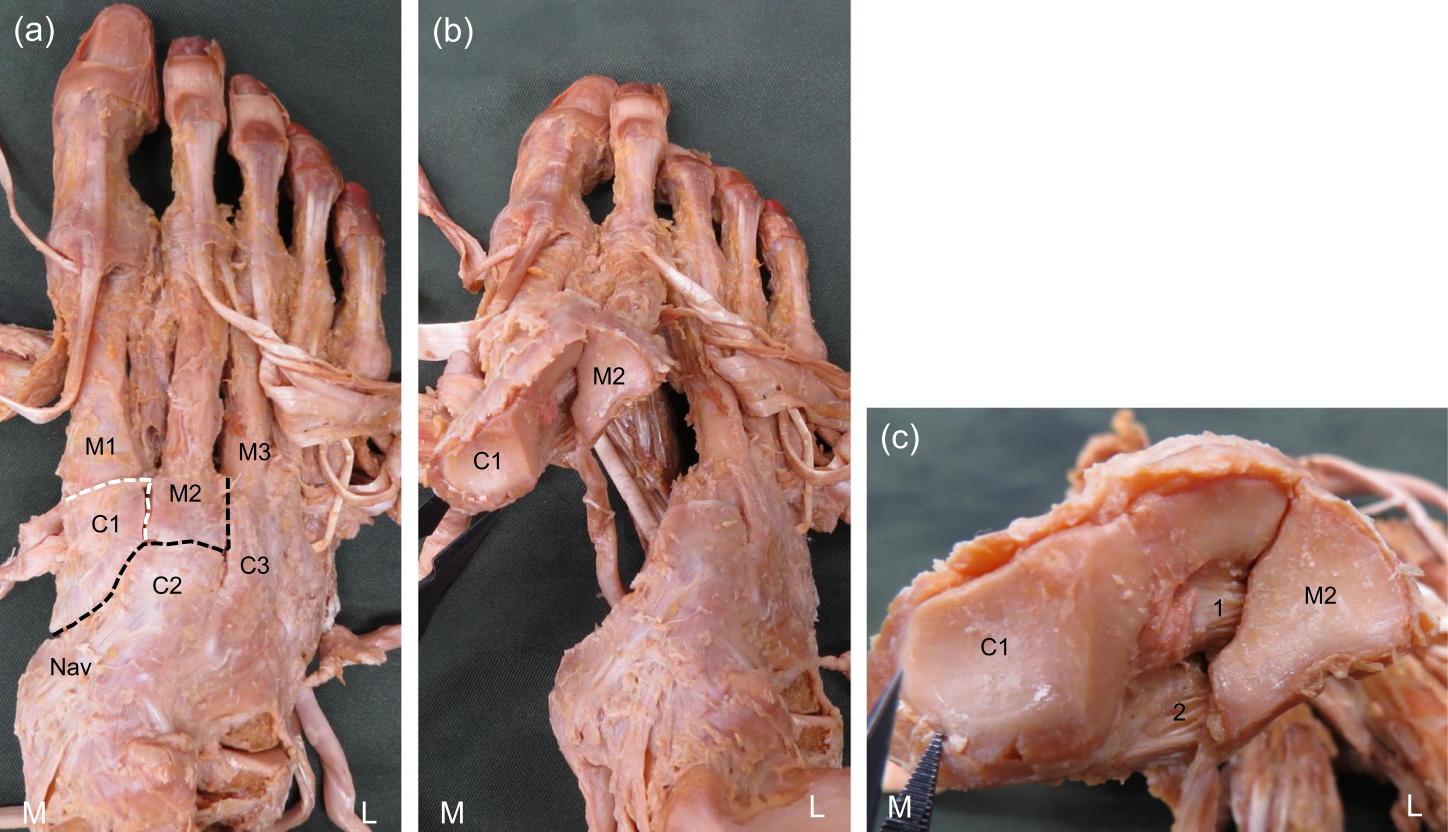
The procedure of the ligament dissection. **a**: Dorsal view of the right foot. **b**: Dorsal proximal view of the right foot. **c**: Articular surface of the first cuneiform and the second metatarsal in the Lisfranc joint. Black dotted line: the parts between navicular bone and the first cuneiform, between the second cuneiform and the second metatarsal, between the second metatarsal and the third metatarsal were separated. White dotted line: the parts between the first metatarsal and first cuneiform, between the first metatarsal and the second metatarsal were separated partially. 1: Lisfranc ligament, 2: cuneiform 1-metatarsal 2&3 plantar ligament, L: lateral, M: medial, C1: first cuneiform, C2: second cuneiform, C3: third cuneiform, M1: first metatarsal, M2: second metatarsal, M3: third metatarsal, Nav: navicular

In the ligament dissection procedure, isolated specimens of the lower leg were prepared by first cutting them off 10 cm above the knee, and the skin, subcutaneous tissue, and crural fascia were then removed. From the plantar and dorsal side, to dissect the Lisfranc ligament and the CMPL, parts between navicular bone and the C1, between the second cuneiform (C2) and M2, between M2 and the third cuneiform (C3), and between M2 and M3 were separated. And parts between M1 and C1, between C1 and M2 were separated partially. In addition, C1 and M2 were removed to the dorsal side. In the classification method, the two criteria were used for defining the difference between the Lisfranc ligament and the CMPL. The two criteria were to determine whether the origin was the same and whether the fiber bundle could be completely separated into a number of fiber bundles (Fig. 1).

**Fig. 2:**
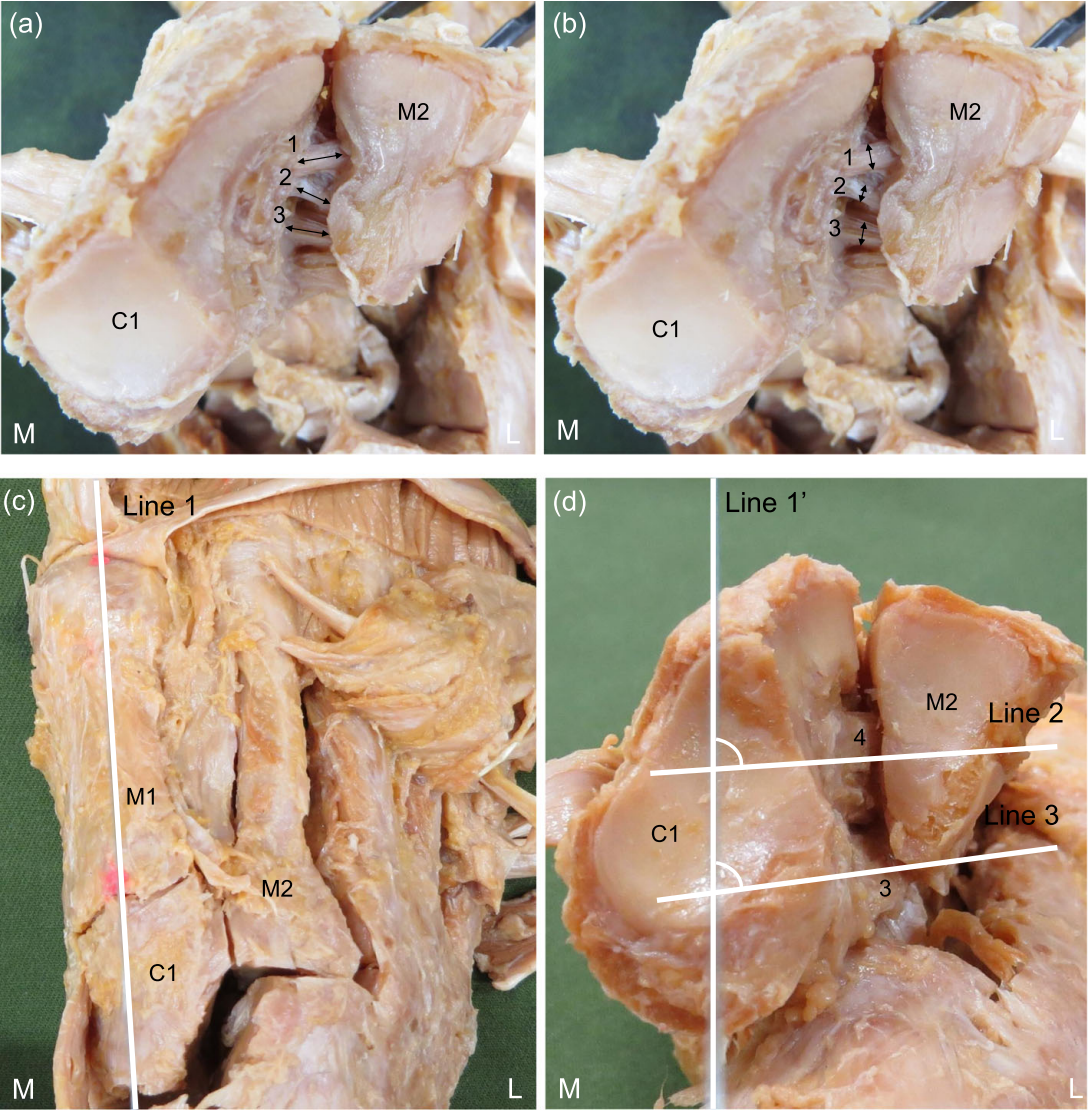
The method for measuring the morphological characteristics of the Lisfranc ligament and the cuneiform 1-metatarsal 2&3 plantar ligament. **a**: The measurement site of the fiber bundle length at articular surface of the first cuneiform and the second metatarsal in the Lisfranc joint. **b**: The measurement site of the fiber bundle width at articular surface of the first cuneiform and the second metatarsal in the Lisfranc joint. **c**: Dorsal view of the right foot. **d**: The method for measuring fiber bundle angle of the ligament at articular surface of the first cuneiform and the second metatarsal in the Lisfranc joint. Line 1: the long axis of the first metatarsal (line connecting the midpoints of the width of the distal and proximal joint surfaces). Line 1′: Line projected onto Line 1 on the articular surface of the first cuneiform in the Lisfranc joint. Line 2: Central portion of the fiber bundle of the Lisfranc ligament. Line 3: Central portion of the fiber bundle of the cuneiform 1-metatarsal 2&3 plantar ligament . 1: the superior fiber bundle of the Lisfranc ligament, 2: the inferior fiber bundle of the Lisfranc ligament, 3: the cuneiform 1-metatarsal 2&3 plantar ligament, 4: Lisfranc ligament, L: lateral, M: medial, C1: first cuneiform, M1: first metatarsal, M2: second metatarsal

Fiber bundle length, fiber bundle width, fiber bundle thickness, and fiber bundle angle were measured for the Lisfranc ligament and the CMPL. The fiber bundle length, fiber bundle width, and fiber bundle thickness were measured in the central portions of the Lisfranc ligament and the CMPL using calipers (Digital Caliper, Shinwa, Niigata, Japan). For measurement of the fiber bundle angle, the long axis of the first metatarsal (line connecting the midpoints of the width of the distal and proximal joint surfaces; Line 1) was first measured [13], and Line 1 was projected on the articular surface of C1 in the Lisfranc joint (Line 1′). The angle between Line 1′ and the fiber bundle was measured using a goniometer (Goniometer, Nishikawa, Tokyo, Japan) (Fig. 2). All measurements were made by the same examiner, with each site measured three times, and the mean value and standard deviation were then calculated. This study examined the intra-rater reliability of morphological characteristics, with retesting performed at an interval of 3–7 days.

### Statistical analysis

Statistical analyses were performed using SPSS (version 24.0, SPSS Japan Inc., Tokyo, Japan). Intersession measurement reliability was assessed using the intraclass correlation coefficient (ICC) (1, 3). The minimal detectable difference at the 95% (MDD95%) confidence interval was calculated as follows [16]: MDD95% = z × SEM × √2, where z = 1.96 and standard error of measurement (SEM) = SD√(1 − ICC). The chi-squared test was used for comparisons between men and women and between right and left in the classifications based on differences in the type. Comparisons of fiber bundle length, fiber bundle width, fiber bundle thickness, and fiber bundle angle between the Lisfranc ligament and the CMPL were made with a paired *t*-test. The level of significance was taken to be 5%.

## Results

### Intra-rater reliabilities and MDD95% values of morphological characteristics

The ICC (1, 3) of the measurement of morphological characteristics by type was 0.90–0.98 (Table 1). In this study, measurement of the morphological characteristics showed almost perfect reliability, consistent with the results of a previous study [17].

**Table 1:** Intra-rater reliabilities and MDD95% values of morphological characteristics for Type **I**

		ICC (1,3)	MDD_95%_
Lisfranc Ligament	Length (mm)	0.98	0.51
	Width (mm)	0.97	1.21
	Thickness (mm)	0.94	0.67
	Angle (°)	0.96	4.22
Cuneiform 1-metatarsal 2&3 plantar ligament	Length (mm)	0.96	0.88
	Width (mm)	0.96	0.97
	Thickness (mm)	0.90	0.79
	Angle (°)	0.94	2.67

### Classification of each ligament

**Fig. 3:**
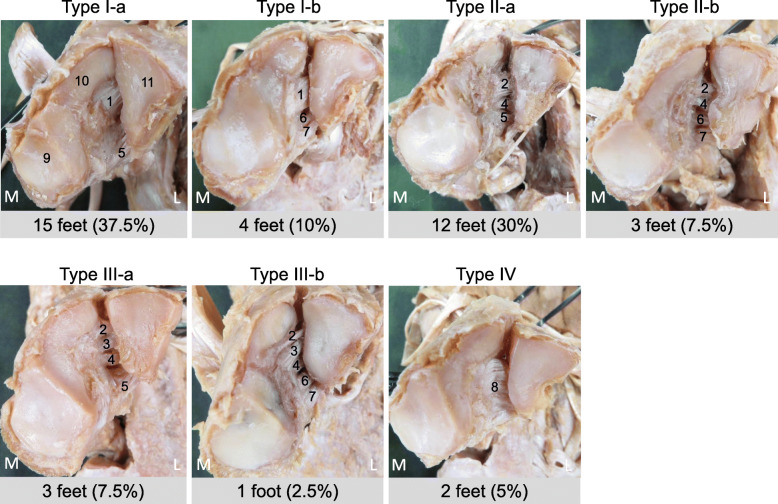
Classification of the Lisfranc ligament and the cuneiform 1-metatarsal 2&3 plantar ligament Type I-a: the Lisfranc ligament is a single fiber bundle, and the cuneiform 1-metatarsal 2&3 plantar ligament is a single fiber bundle. Type I-b: the Lisfranc ligament is a single fiber, and the cuneiform 1-metatarsal 2&3 plantar ligament consists of a superior fiber bundle and an inferior fiber bundle. Type II-a: the Lisfranc ligament consists of a superior fiber bundle and an inferior fiber bundle, and the cuneiform 1-metatarsal 2&3 plantar ligament is a single fiber bundle. Type II-b: the Lisfranc ligament and the cuneiform 1-metatarsal 2&3 plantar ligament consist of a superior fiber bundle and an inferior fiber bundle. Type III-a: the Lisfranc ligament consists of a superior fiber bundle, an intermediate fiber bundle, and an inferior fiber bundle, and the cuneiform 1-metatarsal 2&3 plantar ligament is a single fiber bundle. Type III-b: the Lisfranc ligament consists of a superior fiber bundle, intermediate fiber bundle, and an inferior fiber bundle, and the cuneiform 1-metatarsal 2&3 plantar ligament consists of a superior fiber bundle and an inferior fiber bundle. Type IV: the Lisfranc ligament and t the cuneiform 1-metatarsal 2&3 plantar ligament cannot be separated. 1: the Lisfranc ligament, 2: the superior fiber bundle of the Lisfranc ligament, 3: the intermediate fiber bundle of the Lisfranc ligament, 4: the inferior fiber bundle of the Lisfranc ligament, 5: the cuneiform 1-metatarsal 2&3 plantar ligament, 6: the superior fiber bundle of the plantar ligament, 7: the inferior fiber bundle of the cuneiform 1-metatarsal 2&3 plantar ligament, 8: the Lisfranc ligament and the cuneiform 1-metatarsal 2&3 plantar ligament cannot be separated, 9: the first cuneiform, 10: the first metatarsal, 11: the second metatarsal, L: lateral, M: medial

Using the classification based on differences in the Lisfranc ligament fiber bundles, there were four types: Type I, Type II, Type III, and Type IV. Using the classification based on differences in the CMPL fiber bundles, there were two subgroups in Type I, Type II, and Type III. The types were as follows: Type I-a, the Lisfranc ligament and the CMPL were a single fiber bundle; Type I-b, the Lisfranc ligament was a single fiber bundle and the CMPL consisted of a superior fiber bundle and an inferior fiber bundle; Type II-a, the Lisfranc ligament consisted of a superior fiber bundle and an inferior fiber bundle, and the plantar ligament was a single fiber bundle; Type II-b, the Lisfranc ligament and the CMPL consisted of a superior fiber bundle and an inferior fiber bundle; Type III-a, the Lisfranc ligament consisted of a superior fiber bundle, an intermediate fiber bundle, and an inferior fiber bundle, and the CMPL was a single fiber bundle; Type III-b, the Lisfranc ligament consisted of a superior fiber bundle, an intermediate fiber bundle, and an inferior fiber bundle, and the CMPL consisted of a superior fiber bundle and an inferior fiber bundle; Type IV, the Lisfranc ligament and the CMPL could not be separated. Type I-a was seen in 15 ft (37.5%), Type I-b in 4 ft (10%), Type II-a in 12 ft (30%), Type II-b in 3 ft (7.5%), Type III-a in 3 ft (7.5%), Type III-b in one foot (2.5%), and Type IV in 2 ft (5%) (Fig. 3).

In the comparison between men and women, there were significantly more Type I-a cases in females than in males (*p* < 0.05). There were no significant differences between left and right sides (Table 2).

**Table 2:** Type by sex and side

	Type I-a	Type I-b	Type II-a	Type II-b	Type III-a	Type III-a	Type IV
**Male**	3 (13.6)	3 (13.6)	9 (40.9)	2 (9.1)	2 (9.1)	1 (4.6)	2 (9.1)
**Female**	12 (66.6)*	1 (5.6)	3 (16.6)	(5.6)	1 (5.6)	0 (0)	0 (0)
**Right**	8 (40)	2 (10)	5 (25)	2 (10)	2 (10)	0 (0)	1 (5.0)
**Left**	7 (35)	2 (10)	7 (35)	1 (5.0)	1 (5.0)	1 (5.0)	1 (5.0)
**Total**	15 (37.5)	4 (10)	12 (30)	3 (7.5)	3 (7.5)	1 (2.5)	2 (5.0)

Number (%)

**P < 0.05 (vs Type I-a male)

### Morphological characteristics of each ligament

For the Lisfranc ligament, total fiber bundle length was 5.9 ± 1.5 mm, total fiber bundle width was 5.9 ± 3.2 mm, total fiber bundle thickness was 4.2 ± 1.3 mm, and total fiber bundle angle was 96.4 ± 8.2° (Table 3). For the CMPL, total fiber bundle length was 6.1 ± 1.6 mm, total fiber width was 4.7 ± 1.5 mm, total fiber thickness was 2.2 ± 0.7 mm, and total fiber bundle angle was 81.4 ± 5.2° (Table 4). the CMPL ran from the plantar surface of the first cuneiform to the M2 and M3 in 29 of 40 ft and was attached only to M1 in 11 of 40 ft.

**Table 3:** Morphological characteristics of the Lisfranc ligament

		Length (mm)	Width (mm)	Thickness (mm)	Angle (°)
Type I-a (n: 15)		6.3 ± 1.1	7.9 ± 1.8	4.6 ± 0.9	96.5 ± 6.5
Type I-b (n: 4)		6.6 ± 1.2	8.4 ± 1.0	4.7 ± 0.9	97.2 ± 5.5
Type II-a (n: 12)	Superior fiber bundle	6.4 ± 2.5	4.7 ± 0.6	4.5 ± 1.4	93.6 ± 6.6
	Inferior fiber bundle	5.9 ± 1.2	5.6 ± 2.0	4.7 ± 0.8	99.4 ± 6.2
Average		6.2 ± 1.9	―	4.6 ± 1.1	96.5 ± 6.9
Total		―	10.3 ± 2.0	―	―
Type II-b (n: 3)	Superior fiber bundle	5.9 ± 1.6	3.7 ± 0.5	4.2 ± 1.3	95.3 ± 9.8
	Inferior fiber bundle	5.0 ± 0.7	3.1 ± 0.6	2.3 ± 0.3	111.9 ± 11.2
Average		5.4 ± 1.2	―	3.2 ± 1.4	103.6 ± 13.0
Total		―	6.7 ± 0.1	―	―
Type III-a (n: 3)	Superior fiber bundle	4.2 ± 0.3	2.8 ± 0.8	2.5 ± 0.7	89.1 ± 5.7
	Intermediate fiber bundle	5.4 ± 1.7	3.4 ± 1.1	3.7 ± 1.1	88.3 ± 8.1
	Inferior fiber bundle	5.7 ± 1.2	3.7 ± 0.8	4.3 ± 1.1	102.4 ± 2.5
Average		5.1 ± 1.2	―	3.5 ± 1.2	93.3 ± 8.6
Total		―	9.9 ± 1.6	―	―
Type III-b (n: 1)	Superior fiber bundle	5.6	2.1	1.6	103.7
	Intermediate fiber bundle	5.7	3.2	0.9	98.0
	Inferior fiber bundle	4.5	3.5	4.1	97.7
Average		5.9 ± 1.5	―	4.2 ± 1.3	96.9 ± 7.8
Total		―	5.5 ± 2.3	―	―
Type IV (n: 2)		4.5 ± 0.9	17.8 ± 0.7	4.2 ± 0.1	81.5 ± 3.5
Total		5.9 ± 1.5	5.9 ± 3.2	4.2 ± 1.3	96.4 ± 8.2

**Table 4:** Morphological characteristics of the cuneiform 1-metatarsal 2&3 plantar ligament

		Length (mm)	Width (mm)	Thickness (mm)	Angle (°)
Type I-a		6.0 ± 1.8	5.7 ± 1.3	2.3 ± 0.8	79.5 ± 3.7
Type I-b	Superior fiber bundle	5.1 ± 0.5	3.2 ± 1.0	2.0 ± 0.8	80.9 ± 6.3
	Inferior fiber bundle	7.5 ± 0.8	3.0 ± 0.5	2.6 ± 1.0	78.1 ± 6.5
Average		6.3 ± 1.4	―	2.3 ± 0.9	79.5 ± 6.1
Total		―	6.2 ± 0.8	―	―
Type II-a		6.6 ± 1.4	4.7 ± 1.2	2.2 ± 0.8	81.7 ± 4.8
Type II-b	Superior fiber bundle	4.4 ± 0.3	3.5 ± 0.6	1.5 ± 0.3	86.7 ± 4.8
	Inferior fiber bundle	5.6 ± 0.5	4.4 ± 0.4	1.6 ± 0.2	89 ± 5.2
Average		5.0 ± 0.4	―	1.6 ± 0.3	87.8 ± 4.7
Total		―	7.9 ± 0.6	―	―
Type III-a		6.0 ± 2.1	6.2 ± 2.2	2.1 ± 0.2	82.7 ± 5.5
Type III-b	Superior fiber bundle	5.5	3.5	2.5	80.7
	Inferior fiber bundle	7.2	3.4	1.9	80.3
Average		6.3 ± 1.2		2.2 ± 0.4	80.5 ± 0.2
Total			6.9		
Total		6.1 ± 1.6	4.7 ± 1.5	2.2 ± 0.7	81.4 ± 5.2

The Lisfranc ligament was significantly larger than the CMPL in total fiber bundle width (*p* < 0.05), total fiber bundle thickness(*p* < 0.05), and total fiber bundle angle (*p* < 0.05).

## Discussion

This study elucidated the morphological characteristics of the Lisfranc ligament and the CMPL in Japanese cadavers. To the best of our knowledge, there have been no detailed anatomical studies of these ligaments like the present study.

The classification based on differences in the Lisfranc ligament and the CMPL was Type I-a in 15 ft (37.5%), Type I-b in 4 ft (10%), Type II-a in 12 ft (30%), Type II-b in 3 ft (7.5%), Type III-a in 3 ft (7.5%), Type III-b in one foot (2.5%), and Type IV in 2 ft (5%). Previous anatomical studies reported that the Lisfranc ligament has a single fiber bundle [10], two fiber bundles (single fiber bundle in 73%, two fiber bundles in 27%) [12], and four fiber bundles (17 cases of one, 45 cases of two, 14 cases of four) [11], that the CMPL has varied directionality, and it divides into three directions depending on ligament morphology: linear in 32 cases, Y-shaped in 32 cases, V-shaped in 8 cases, and unclassified in 2 cases [11]. Therefore, no consensus has been obtained, and there are differences from the results of the present study. The reason for the differences was thought to be that it is difficult to distinguish between the Lisfranc ligament and the CMPL. The origin of the CMPL is defined as the C1 sublateral surface [10] or the C1 lateral surface [15], which is adjacent to the origin of the Lisfranc ligament. Therefore, it is possible that differences in the views of the Lisfranc ligament and the CMPL may occur between studies. In the present study, classification was performed depending on whether the origin of the Lisfranc ligament and the CMPL clearly differed from each other as a criterion.

Regarding sex differences, Type I-a was significantly more common in females than males. In previous studies, sex differences were not sufficiently investigated. It will be necessary to further investigate the cause for the sex difference in the future.

In the present study, the morphological features of the Lisfranc ligament and the CMPL were obvious. In previous studies of the Lisfranc ligament, it was reported that the fiber bundle length was 8.02 ± 1.5 mm [13], 9.17 ± 1.5 mm (6.6–10.95) [14], and 33.7 ± 0.8 mm (2.2–3.1) [15]. The fiber bundle width was 2.53 ± 0.61 mm [13], 5.21 ± 1.28 mm (3.75–7.55) [14], and 12.5 ± 2.8 mm (8.7–18.1) [15]. The bundle thickness was 5.4 ± 1.4 mm (3.1–8.1) [15], 6.9 ± 1.28 mm (5–9.1) [14], 6.96 ± 1.01 mm [13], and 7.68 ± 1.25 mm [16]. For the plantar ligament, the bundle thickness was 3.25 ± 0.97 mm [16]. Therefore, no consensus has been obtained, and there are differences from the results of the present study. The reason for the differences was thought to be that it is difficult to distinguish between the Lisfranc ligament and the CMPL. In addition, it was considered that there were differences in measurement methods and in the number of samples. Hirano et al. [11] used a caliper for fixed cadavers (*N* = 78), Kura et al. [15] used a caliper for fresh-frozen cadavers (*N* = 12), Johnson et al. [16] used calipers for fresh-frozen cadavers (*N* = 20), Castro et al. [14] used MRI for an in vivo study (*N* = 10), and Ablimit et al. [13] used MRI for an in vivo study (*N* = 60).

And, the morphological characteristics of each type were also obvious. Although no statistical analysis was performed, each type of the morphological characteristics did not differ significantly, suggesting that the difference in the number of fiber bundles in each ligament may not be involved in the stability of the Lisfranc joint.

In comparisons of morphological features between the Lisfranc ligament and the CMPL, the Lisfranc ligament was significantly larger than the CMPL in total fiber bundle width, total fiber bundle thickness, and total fiber bundle angle. In previous studies, Kura et al. [15] found the thicker, more plantar ward ligament that they described as the Lisfranc ligament to be stronger than the thin dorsal ligament. De Palma et al. [10] found the interosseous ligament (Lisfranc ligament) to be the thickest compared with the dorsal and the CMPL. Therefore, the present study supported the previous study. Regarding total fiber bundle angle, in the biomechanical study using fresh-frozen cadavers, amputation of the Lisfranc ligament and the CMPL was necessary to cause instability of the Lisfranc joint (C1-M2 joint and C2-M2 joint) [12]. Therefore, both ligaments may stabilize the Lisfranc joint.

The limitation of this study is that only the morphological features of the Lisfranc ligament and the CMPL were examined using fixed cadavers. What the relationship is between Lisfranc joint injury and ligament type in vivo remains unknown. Therefore, an in vivo study using ultrasound examination is needed in the future.

## Conclusions

This study elucidated the morphological characteristics of the Lisfranc ligament and the CMPL in Japanese cadavers. It was found that the Lisfranc ligament had up to 3 fiber bundles and the CMPL had one or two fiber bundles, with classification into four types and two subgroups. Based on the results of the present study, it is necessary to examine the relationship between types and injuries in vivo.

## Data Availability

The data that support the findings of this study are available from the corresponding author upon reasonable request.

## References

[1] Lewis JS (2016). Lisfranc Injuries in the Athlete. Foot Ankle Int.

[2] Aitken AP (1963). Dislocations of the tarsometatarsal joint. J Bone Joint Surg Am.

[3] Rajapakse B (2006). A single surgeon's experience of treatment of Lisfranc joint injuries. Injury..

[4] Goossens M, et al. Lisfranc's fracture-dislocations: etiology, radiology, and results of treatment. A review of 20 cases. Clin Orthop Relat Res. 1983;176:154. 6851319

[5] Moracia-Ochagavia I (2019). Lisfranc fracture-dislocations: current management. EFORT Open Rev.

[6] Myerson MS (1986). Fracture dislocations of the tarsometatarsal joints: end results correlated with pathology and treatment. Foot Ankle.

[7] Desmond EA (2006). Current concepts review: Lisfranc injuries. Foot Ankle Int.

[8] Kaar S (2007). Lisfranc joint displacement following sequential ligament sectioning. J Bone Joint Surg Am.

[9] Sappey PC (1888). Traite d’Anatomie Descritive.

[10] de Palma L (1997). Anatomy of the Lisfranc joint complex. Foot Ankle Int.

[11] Hirano T (2013). Anatomical considerations for reconstruction of the Lisfranc ligament. J Orthop Sci.

[12] Panchbhavi VK (2013). Three-dimensional, digital, and gross anatomy of the Lisfranc ligament. Foot Ankle Int.

[13] Ablimit A (2018). Magnetic resonance imaging of the Lisfranc ligament. J Orthop Surg Res.

[14] Castro M (2010). Lisfranc joint ligamentous complex: MRI with anatomic correlation in cadavers. AJR Am J Roentgenol.

[15] Kura H (2001). Mechanical behavior of the Lisfranc and dorsal cuneometatarsal ligaments: in vitro biomechanical study. J Orthop Trauma.

[16] Johnson A (2008). Anatomy of the lisfranc ligament. Foot Ankle Spec.

[17] Landis JR (1977). The measurement of observer agreement for categorical data. Biometrics..

